# Analysis of the Quantitative Evaluation of the Public Medical and Health System Costs During Pandemic Governance: Investigation Based on COVID-19

**DOI:** 10.3389/fpubh.2022.942043

**Published:** 2022-07-13

**Authors:** Xintao Li, Zaisheng Zhang, Li Liu, Tongshun Cheng, Gang Liu

**Affiliations:** ^1^Zhou Enlai School of Government, Nankai University, Tianjin, China; ^2^Chinese Government and Policy Joint Research Center, Nankai University, Tianjin, China; ^3^Tianjin Federation of Social Science, Tianjin, China; ^4^School of Tourism, Hainan University, Haikou, China

**Keywords:** pandemic governance, public medical and health system costs, quantitative evaluation, the grounded theory, the combinational evaluation of subjective and objective method

## Abstract

It is of great reference significance for broadening the research perspective of pandemic governance, improving the efficiency of pandemic governance and the credibility of the government, to scientifically measure and analyze the public medical and health system costs. This article takes the typical case “pandemic prevention and control event of S city, China” as the research background. First, the concept of public medical and health system costs during pandemic governance is defined. Then, the public medical and health system costs are embedded into the pandemic governance system, and the generation process of the public medical and health system costs in the actual situation are investigated. Furthermore, through in-depth interview, multi-case grounded theory and fuzzy subordinate function analysis, the scientific construction of the public medical and health system cost index system are completed. Finally, based on G1 method/entropy method combined with weighting and fuzzy comprehensive evaluation method, the public medical and health system costs of the pandemic prevention and control events of S city is measured. The results show the following: (1) it is important that good single dimensions and reliable indicators are embodied in the public medical and health system costs scale. Among them, the behavioral public medical and health system costs of the masses is the largest proportion of all indicators; (2) after the pandemic prevention and control event is over, the public medical and health system cost are difficult to repair, and some lagging ideas and behaviors shown by local governments lead to a continuous expansion of the public medical and health system costs associated with pandemic governance; and (3) local governments should not conceal information asymmetry. Instead, local governments should give greater freedom to other actors to deal with pandemic governance, and governance entities should cooperate with each other. This will mitigate the effect of public medical and health system costs. Corresponding policy recommendations are proposed.

## Introduction

It can be said that the prevention and control of COVID-19 is a centralized test of the emergency management system and governance capacity of various countries. Although China's current pandemic prevention and control situation continues to show good momentum, the pandemic prevention and control process has exposed a series of problems: public opinion risk monitoring and publicity guidance lag, and the public psychological intervention mechanism is absent (1). There are also some prominent problems. For example, the organizational and institutional systems of pandemic prevention and control are not fully connected and coordinated, and the various prevention and control agencies remain relatively independent in their operations. It should be noted that many public functions involved in pandemic prevention and control are not fully divided, and problems of overlapping or lagging gaps still exist to varying degrees (2). In addition, the partial defects and implementation errors of the emergency prevention and control system have resulted in an unbalanced distribution of benefits and costs, prominent contradictions between economic development and pandemic prevention and control, and frequent conflicts of rights protection incidents in pandemic prevention and control. These phenomena will reduce the credibility of a government and the public satisfaction, resulting in an unnecessary waste of the public medical and health system resources (intangible resources) (3). The government must increase the public medical and health system resource inputs to achieve pandemic governance goals, which constitutes the public medical and health system costs during pandemic governance. The process of consuming public medical and health system resources related to pandemic concerns is accompanied by complex issues, such as ethics, government and citizen behavior, and legitimacy, and it difficult for traditional governance approaches to effectively deal with multiple conflicting interests in pandemic governance (4). Medical system costs for pandemic prevention and control involve complex issues such as public demand and political legitimacy, and the effectiveness of governance depends on public recognition, participation, and support (5). Public health is a major system issue. Therefore, the study of the public medical and health system costs associated with pandemic governance problems requires multidisciplinary theories and methods, such as public crisis management, government economics, and social security, but it also should pay attention to obtaining control instruments from a multi-disciplinary and multiple-research perspective.

## Definition of Concepts

The pandemic governance issues have risen to the level of system issues, and has become an important part of the governance system of many countries. The introduction of political science, public administration, and economics in pandemic governance are conducive to help the government to determine an effective and precise path with respect to pandemic control precisely and effectively. With the increasing diversity and complexity of public affairs, the importance of the cost of system is a concept that has emerged repeatedly. David Easton (6) proposed that, for a public system to function properly, it must have some resources to serve as a driving force and foundation (6). Harold D. Lasswell (7) and Dennis Wrong (8) subsequently continued to deepen the interpretation of the concept of system resources, arguing that elements such as the institutional mechanisms, organization and culture are a means to influence system objects that can be constantly consumed and lost (7, 8). The government needs to have and consume certain public medical and health system resources, and thus reflects the value of its existence and achieves its pandemic governance goals (9). It can be divided into “tangible resources” (e.g., economic resources), which are materially consumed, and “intangible resources” (e.g., public medical and health system resources), due to the hidden characteristic of intangible costs, they are often ignored by the government, examples of which include exist objectively organizational resources, and the system of democracy by the government (10). Under the coercive character of the governmental system, the institutional discrimination, uneven distribution of governmental public goods supplies and benefits, and unfair procedures will trigger protests from various interest entities (11). Therefore, a government that values and effectively uses intangible resources will appear more stable in its legitimacy base and public security (12).

In the process of pandemic prevention and control, various complex and comprehensive cost containment strategies are being developed across the most regions tailored to his national needs (13). The governance of the pandemic is regarded as a public choice process in the decision-making of local governments. Local governments as Rational Economic Man and agents have the necessity to provide the necessary public products for the public to carry out the pandemic prevention and control. Various “transaction costs” will generate in the process of governance, so the “care” for all stakeholders fairly cannot be realized. However, the design of the public health and medical system will be influenced by all stakeholders through various channels. The disturbance of the optimality of the public health and medical system, the consumption of the limited system resources in the pandemic prevention and control, and the generation of certain social risks will be caused by the interest expression and demands of all stakeholders. The reality and system defects promote the formation of the social risk. Once it is embedded in the social structure, the elimination of its substantive risk will not be realized, and the possibility of evolution or even mutation will exist. The external factors such as the media will easily influence the public's perception in the pandemic prevention and control. No matter it is scientific and reasonable or not, the public will firstly “amplify the problem” and actively pursue risks instead of considering the outcome, since the greater benefits will be brought to them. What is worse is that the potential risks of pandemic prevention and control derived from it will evolve into social risks.

The local government, as the institution that ultimately allocates resources in pandemic, needs to comprehend considerable amounts of information and conduct a scientific analysis of pandemic governance when governing the pandemic, and convene relevant experts to discuss and scientifically evaluate the feasibility and operability of various options (14). It also needs to assess the risks, public opinion, and benefits associated with implementing a certain option (15). When the validated decisions are put into practice, the waste and loss of public medical and health system resources caused by unscientific decisions can be avoided. In addition, the interests of the public and other governance entities have led to the selective allocation of public medical goods supply and delicacy domination of medical system resources, which makes neglected medical system resources constantly visible with the expression of public demands (16), which has meant that public medical and health system resources are constantly associated with the expression of public demands or the occurrence of protests. With the development of democratization and the increase in public awareness of pandemic rights and participation, the public's understanding of public medical and health system resources has gradually changed to materialization (17). Based on the above analysis, the public medical and health system costs are the system resources consumed by the government or others in the process of exercising political power and taking political actions in order to achieve pandemic governance and obtain public medical and health governance performance, as well as the public medical and health system resources to be borne by society and organizations. The consumption of these system resources will have various impacts on the society. The public medical and health system costs are intangible and scarce costs, which are difficult to manifest before accumulating to a certain extent and have the hidden characteristic, which contain the following elements:

(1) The public medical and health institutional policy costs (institutional policy costs for short). Such as institutional laws and policies and regulations in the institutional system of pandemic governance (18).
(2) The public medical and health system organizational costs (system organizational costs for short). Such as the government, social organizations, and relevant staff, etc. which together form the system organization that guarantees the effective functioning of pandemic governance (19).
(3) The public medical and health social perceptive costs (social perceptive costs for short). Such as the publicity and education costs, ideals beliefs, and social psychology, which could make the long-term behavior of social entities produce pandemic governance identity (20).
(4) The public medical and health mass behavioral costs (mass behavioral costs for short). The treatment of COVID-19 control mass events is a complex and long-term systemic project that requires long and uninterrupted public participation and support (21). The public satisfaction, identity, and trust are also important.

## Literature Review

At present, effectively preventing and controlling the pandemic remains the focus of most management scholars. First, in the analysis of the efficiency of pandemic prevention and control, scholars have generally turned to the analysis of the institutional mechanisms and participation factors, such as the public's participation in pandemic prevention and control, the distribution of interests among medical bodies, and the public medical and health system in community areas. All these affect the degree of prevention and control of the pandemic, and problems with pandemic prevention and control in the region should be presented in various states such as social perception (22–24). Second, in terms of prevention and control approaches, scholars believe that media plays important role in the pandemic prevention and control process at the present stage. The current pandemic prevention and control emergency system is slightly weak, the management system is not perfect, and the technical support system is underdeveloped. Only by establishing a multi-center pandemic prevention and control model can the pandemic be truly controlled. The prevention and control of the pandemic should be carried out through effective coordination between regional governments and departments. It is necessary to improve the performance incentive mechanism of local pandemic prevention and control to improve the responsibility and attention of local officials, while actively carrying out audits of the whole process and policy evaluations of pandemic prevention and control. This will give full play to the role of elites in pandemic prevention and control (25–27). Third, there are various research methods for pandemic prevention and control, according to the multiple participants involved. In order to optimize current prevention and control policies and measures, scholars often use collaborative degree models, regression models, social network models, evolutionary game models, and simultaneous equations to analyze the relationship between government, society, enterprise, and the public (28–30). Fourth, scholars actively participate in the exploration of risk management in pandemic prevention and control, and constantly reiterate the importance of risk source identification, emergency resource assessments, monitoring and early warning, advance emergency preparedness, inter-departmental emergency coordination, information disclosure, and public opinion response in pandemic prevention and control. They focus on the whole process of emergency management and network collaboration between departments, and aim to reform the emergency management organization system (31–33). Researchers have conducted in-depth discussions on the mechanisms and paths of pandemic prevention and control, the economic costs of the government, and the issues of governance and supervision, with many research achievements. It is obvious that these studies have not taken into account the medical system costs of pandemic prevention and control, and they lack of an understanding of its importance and necessity. In terms of methods, research on the evolution, perception behavior, and other aspects of the participants in each stage of the pandemic prevention and control node is still lagging behind. Moreover, local government departments tend to ignore medical system costs, influenced by the ideas of “Promotion–Accountability,” “Growth First” or “Rational Economic Man.” The governments pointed out many times in the prevention and control of COVID-19 that as the situation of pandemic prevention and control is constantly changing, new situations and new problems arise (34). Based on past experience, the spread of the epidemic will have an impact on the normal operation of the economy, and then it will evolve into an important exogenous factor affecting economic growth. Under the impact of the epidemic, the behavior patterns of various entities have undergone drastic adjustments (35). During the protests against and the death of patients in certain sections of Asia, the public first defended their rights and expressed their interests by reasonably expressing their demands. However, due to mishandling by local governments, the low governance capacity of some public officials, and individual local departments who were fighting for themselves and maintaining stability first, the demands of the public could not be effectively resolved. Therefore, scientifically controlling medical system cost consumption in the prevention and control of the pandemic is a major issue that the local government (hereafter referring to government) must face. Therefore, when considering pandemic governance, attention should be paid to the excavation, measurement, and effective use of public medical and health system costs, and as variables for evaluating pandemic governance efficiency. Because attention to these issues will highlight deep-seated and hidden problems associated with pandemic governance, which will substantially enhance the quality and effectiveness of pandemic governance (14). The study contributes to broadening the research perspective of pandemic governance.

However, neither academics nor political circles have established a targeted and systematic evaluation index system and measurement tools for the public medical and health system costs. Most researchers have analyzed the economic cost of pandemic governance from the aspect of “cost-benefit.” The study and measurement of the public medical and health system costs of pandemic governance are still in infancy, and there is a lack of field research and empirical analysis in the form of scale development and testing. Therefore, this study mapped public medical and health system cost attrition to a specific field of governmental governance process by combining typical case examples of pandemic governance in China. At the same time, based on the concept and characteristics of public medical and health system costs, this study adopted multi-case rooting theory and fuzzy affiliation evaluation to complete the scientific construction of a public medical and health system cost index system, which turned the public medical and health system cost into an observable variable or outcome variable. In a case study, the public medical and health system cost in S city was measured using the G1/Entropy combination weighting and the fuzzy comprehensive evaluation method, so that the government could perceive the public medical and health system costs in time and introduce or adjust relevant policies and governance programs in a timely and scientific manner.

## Construction of the Measurement Index System

The construction of the public medical and health system costs index system is a complex, systematic project, and the selection of a suitable method is a key part of system construction. It is also important to ensure the scientific basis, operability, and measurement quality of the index system. In addition, it is difficult to quantitatively measure the public medical and health system costs using a single, quantitative, statistical instrument because of the difficulties associated with a quantitative analysis, such as public approval and support. However, since the public medical and health system costs have measurability, they can be assessed using macro data analyses, social surveys, public opinion support and satisfaction tests, public opinion observation, and other measurement tools (36, 37). In view of this, the construction of public medical and health system costs index system is based on the following principles: the first is the principle of operability. It should be guided by relevant theories and existing cases about the public medical and health system costs to ensure that the measurement indicator system is logical and reasonable, that the evaluation is comprehensive and credible, and to make it operational and reliable. The second is the principle of comparability. The selection of the indicator system involves a comparison of different regions, different time periods, and different stages of the indicator system with each other. Therefore, when establishing the indicators, comparisons need to be made between regions or different time periods within the same region so that the indicators are representative and typical. The third is the principle of hierarchy. The public medical and health system costs involve a variety of structural linkages. The intersection of different fields and interdisciplinary synergies mean that the design of the indicator system needs to be coordinated in terms of classification standards and caliber of indicators so that the operational procedures of the evaluation objectives are understood at a general level, the main and secondary indicators should be clarified, the indicators should be interlinked and complementary to each other, and special and qualitative indicators should be scientifically based so that the statistical data are valid (38). The fourth is the principle of combining subjectivity and objectivity. Qualitative or quantitative indicators with clear conclusions should be identified through testing, surveys, and reviews (39), and the indicators should improve the collection and gathering of specific data so that the indicator system can objectively and truly reflect the consumption of public medical and health system costs in order to ensure the validity of the evaluation.

Based on the above principles, this study selected the pandemic governance case in S city that lasted for nearly 2 years, that provided the largest amount of information about the measurement of the public medical and health system costs in order to facilitate field research, data collection and in-depth analysis. The reasons for choosing this case were as follows: (1) the whole pandemic governance event was longitudinal in time. It contained a complete and clear evolution of the public medical and health system costs. (2) It could meet the purpose and requirements of the study. In the case of pandemic governance, the government, enterprises, social organizations (media), the public, and other governance subjects could interact in a hierarchical ladder, and the various elements of the public medical and health system costs can be fully displayed. (3) The selected pandemic governance case included a wide range of recent public opinion and social influence, and the information available for investigation and research should cover the issues to be studied as much as possible. The S city was the main sample case, and the seven selected typical cases of pandemic governance were randomly divided into two groups, one of which was used to extract the measurements and the other to test the measurements (Table 1).

**Table 1:** Sample cases of the public medical and health system costs studies on pandemic governance.

**Case sample name**	**Department**	**Data collection method**	**Duration (hour)**	**Word count**
Pandemic governance events in Wuhan City (2020) Screening	Academy of Social Sciences	In-depth interviews	1	1,452
Pandemic governance events in Mudanjiang City (2020) Screening	High School	In-depth interviews	1	1,533
Pandemic governance events in Harbin City (2021) Screening	Research Institute	Symposium	1	1,769
Pandemic governance events in Xi'an City (2021) Screening	Government Departments	Symposium	1.5	2,667
Pandemic governance events in Xingtai City (2020) Screening	College	In-depth interviews	1.5	2,410
Pandemic governance events in Shenyang City (2021) Inspection	College	In-depth interviews	2	3,158
Pandemic governance events in Tonghua City (2021) Inspection	Government Departments	Symposium	2	3,477

In order to further develop the measurement index system, field research and data collection were carried out in strict accordance with a set plan and procedures: Step 1—before beginning the field research, a large amount of information was collected and case screening was conducted to identify the objects to be researched. This included using the Internet (search engines, WeChat, Tik-Tok, posting forums, microblogs, QICQ groups, etc.), databases (CNKI, WEB-SCI, SCI-Direct), and the telephone to collect relevant secondary information (see Table 2). Step 2—the research plan was formulated and the field research was conducted. In order to prevent some unnecessary factors from interfering with the research, such as interference by the “gatekeeper,”^1^ the role of the “insider” was adopted. The participant was observed and allowed to interact with the respondents and interviewers in a participatory manner so that they relaxed their vigilance and lowered their guard, and to ensure that the objectivity and authenticity of the research. Step 3—The interviews were recorded, with the consent of the interviewees. Face-to-face in-depth interviews and symposiums, as well as a combination of semi-structured and focus group interviews, were used. This facilitated the organization and coding of the original interview data (Table 3), and relevant primary data were collected after interviewing experts, government public officials, and members of social organizations who had studied and participated in, and paid attention to the seven typical cases selected (14 interviews were conducted, and about 16,000 words from interview scripts were compiled) (Table 1). Step 4—With the help of ATLAS.ti8 software, the literature and research data were summarized and coded. A system for the public medical and health system costs index system was derived by assigning the “the public medical and health system costs concept” to the data.

**Table 2:** Research data collection.

**Access to information**	**Source**	**Main contents**	**Quantity**	**Date of publication**
Baidu/Google: news, reviews, interviews (D1)	Sina-Blog, Phoenix Finance, S-Government.com, etc.	Development background and the practices of government, nodal events, media commentary, expert views	116	2020.3–2020.7
CNKI, WEB-SCI, SCI-Direct (D2)	Periodicals	Pandemic governance events, etc.	372	2020.11–2021.3
WeChat/Tik Tok/Posting forum/ QICQ group (D3)	Silent Spring public website, S city protection public website, S city's posting bar, Y people in S City and other QICQ groups	Attitudes of local governments and enterprises, official reports, strategies and behaviors, media-related analyses, perception of risks, etc.	719	2020.11–2021.4
Collected through field observation	Villagers provide, observation records	Observation records, research reports, local materials, etc.	15	2020.3, 2021.1

**Table 3:** Basic information about the interview.

**Interviewees (Coding)**	**The form of interview**	**The length of interview (duration)**	**Length of the interview (word count)**
Villager A1, A2, A3 of S City	Semi structured interview	3 h	3,010
Villager B1, B2, B3 of S City	Semi structured interview	1.5 h	2,671
Citizen F1 of S City	In-depth interview	2 h	2,246
Citizen X1, X2 of S City	Semi structured interview	2 h	2,870
Staff C of S municipal government Staff member W of Center for Disease Control and Prevention	Symposium and interview focus groups	3 h	5,742

### Open Coding

With the help of ATLAS.ti8 analysis software, the primary textual information from several typical cases was collected and transcribed to be decomposed. In order to check the authenticity, completeness, and accuracy of the sources of the interview recordings and research data, the data were labeled (zx), formed into primary codes (ZZx), codes (Zx), and finally into open-ended codes (Dx). The same or similar elements existing in the primary information and the primary indicator system were retained, and the more occurrences, the more meaningful the phenomenon represented by the label. A total of 191 primary codes, 87 codes, and 25 open codes were collated and formed, and the relationships between the open codes for the the public medical and health system costs were juxtaposed with each other and their functions, and processes were obtained (see Table 4 for the open code formation process). After coding, a total of 38 open codes and 10 main axis codes were obtained for the public medical and health system costs measurement index system. An initial system (consisting of 1 primary indicator, 4 secondary indicators, 10 tertiary indicators, and 38 quaternary indicators) was established that was decomposable, independent, comprehensive, and easy to operate.

**Table 4:** Example of the open coding process for the public medical and health system costs indicator system (excerpt).

**Open coding (conceptualization)**	**Code (Z_**x**_)**	**Primary code (ZZ_**x**_)**	**Original information about the case (Z_**x**_)**
Degree of effectiveness of enacting policies and regulations at certain phase of the pandemic governance (including regulation, governance measurements, benefit compensation, etc.) (D_1_)	Large number of policies introduced during the period (Z_2_)	Regulation, governance measurements, standards, benefit compensation and other policy measurements continue to improve (ZZ_5_)	Beijing, Tianjin, and Hebei have issued a “Action plan for the epidemic prevention and control measures” and a “Personnel checking and remote management in the city during pandemic governance and control”; Hebei Province has issued a “Notice on Control and management of external personnel”. (Z13)
Degree of closure of telephone/internet complaint cases within the phase of the pandemic governance (D_9_)	Inconsistency in the extent of case handling (Z_18_)	Slow processing speed has led to a decline in credibility (ZZ_43_)	“Public complaints about this incident were handled fairly smoothly.” (Z_41_) “We still have blind spots in supervision, and it is often difficult to monitor so it delays the speed of case completion.” (Z_39_) “I think some of the complaints are making a show whether it is the government or the companies.” (Z_43_)
Degree of public official accountability (D_22_)	The depth of the degree of punishment for accountability (Z_45_)	The heads of government officials, etc. associated with the misconduct of pandemic governance have been prosecuted, suspended, etc. (ZZ_105_)	“The deputy secretary of the party group in X District of T City was removed from his post and the deputy mayor involved was transferred to the judicial authorities.” (Z_103_) “In our research, we found that enterprise “shut down” behavior was spared by some local governments, which is undoubtedly a distorted view of performance.” (Z_104_) “In this pandemic governance, we implemented administrative accountability for 26 government staff, 12 of whom were removed from their posts.” (Z_101_)
Perceived fairness of the interaction (D_24_)	The perceived fairness of government and public response attitude during the process of conflict resolution (Z_52_)	Interpersonal equity refers to the extent to which the public and enterprises are respected and cared for by the government. (ZZ_121_)	“Local governments around Dailian and Shenyang promote the solution of the problem through public announcements of assessments, symposiums, and household visits.” (Z_119_) “I've been kicked around by phone calls and emails.” (Z_56_) “Our governments all convince people to understand the government and invite them to watch the supplies through face-to-face dialogues.” (Z_182_) “The tendency of the government to interact with property owners in a multi-dimensional and proactive manner.” (Z_115_)
Public opinion collection and departmental transfer capability (D_26_)	The transmission, interoperability and sharing of information and materials between departments (Z_56_)	Collect and sort out network public opinion, and report the collected information to other departments, so as to formulate relevant collaborative countermeasures (ZZ_129_)	“Government departments are now establishing an early warning mechanism for online public opinion to understand the real-time development of pandemic governance.” (Z_80_) “The local government is controlling online public opinion in a timely manner, and collaborating with the propaganda and Internet information departments to guide and educate on rumors.” (Z_131_) “Exposure in new media and self-media is higher and the local government does not take note of the information.” (Z_128_)
…	…	…	…

### Associative Codes

The public medical and health system costs indicator system is a multi-level and multi-factor composite structure. Starting with the original concept, this study conceptualized and simplified it by combining previous relevant studies at home and abroad. The system was first divided into two levels: the first level indicator was the total measurement target- public medical and health system costs; and there were four second level indicators, i.e., institutional policy costs, system organizational costs, social perceived costs, and mass behavioral costs. On this basis, the meaning and relationship of each main axis code were reorganized to form four relatively independent core categories and 10 corresponding main axis codes (see Table 5 for details).

**Table 5:** Associative codes.

**Core categories**	**Main axis coding**
The institutional policy costs (B_1_)	Validity of institutional tools (C_1_)
	Validity of system implementation (C_2_)
	Government credibility and implementation (C_3_)
The system organizational costs (B_2_)	Competence level of public officials (C_4_)
	Integrity level of public officials (C_5_)
The social perceptive costs (B_3_)	Perception of social equity (C_6_)
	Effectiveness of public opinion regulation (C_7_)
	Social risk perception (C_8_)
The mass behavioral costs (B_4_)	Implicit participation behavior of the masses (C_9_)
	Explicit participation behavior of the masses (C_10_)

### Indicator Amendments

This study followed the scale development procedures of Dunn et al. (40), Marra et al. (41), and others, and combined qualitative analysis methods with scale development testing methods to design questionnaire items, thus ensuring the scientific and objective nature of the assessment. At the same time, the indicators for the public medical and health system costs were revised to solve the problems associated with a large number of indicators, weak generalizability, vague semantics, lack of operability, and an inability to adapt to changing pandemic situations. First, using the expert survey method, this study selected 32 experts, government officials, and members of social organizations who have been engaged in pandemic governance and public crisis management research fields for many years from the regions where typical cases of pandemic governance have occurred. The initial index system was constituted as an expert screening questionnaire for the public medical and health system costs measurement index system. Through a combination of in-depth interviews and expert symposiums, experts and researchers in relevant fields were invited (from universities, research institutions, government agencies, social organizations, and other institutions) to select the indicators they considered the most important for the public medical and health system costs index based on their own academic knowledge and research experience. The aim was to understand their views and opinions and collect constructive opinions. A total of 32 copies of the expert screening questionnaire were returned, of which 30 were valid, and the questionnaire recovery rate and efficiency rate were above 93%. The basic information statistics for the experts are shown in Table 6(A). After distributing the questionnaires to collect the survey data, this study undertook a redundancy analysis of the information given by the experts, refined the summary, and used fuzzy statistical analysis to eliminate the measurement items with low affiliations to finally determine the formal measurement index system.

Table 6Expert screening results and memberships for each measurement.

**Table:** 

**(A) Expert information statistics**		
**Project**	**Category**	**Proportion**
Gender of the expert	Male	63.3
	Female	36.7
Age	26–29 years old	10
	30–39 years old	20
	40–49 years old	43.3
	Over 50 years old	26.7
Education level	Undergraduate	16.7
	Master	40
	Doctorate	43.3
Work unit	Government agencies	23.3
	Research Institutes	16.7
	Universities	50
	Social organizations and other institutions	10
Title	Primary Title	0
	Intermediate Title	26.7
	Deputy Senior Title	33.3
	Senior title	40

**Table:** 

**(B) Selection results and membership of experts**			
**Four levels of indicators, number of expert tick marks, membership values, retention (yes/no) for the public medical and health system costs**	**Number of expert selections**	**Membership function value**	**Reserved** **(yes/no)**
Degree of effectiveness of policies and regulations enacted during the period (including regulation, governance measures, benefit compensation, etc.) (D_1_)	14	0.47	N
Degree of effectiveness of existing policies and regulations (including regulation, governance measurements, benefit compensation, etc.) (D_2_)	29	0.97	Y
Scientific basis and legitimacy of pandemic governance policy tools (including the degree of expert validation, assessment system, etc.) (D_3_)	29	0.97	Y
Completeness of the emergency prevention and control system (mechanism) (D_4_)	27	0.9	Y
Consistency of system policy and government implementation (D_5_)	28	0.93	Y
Degree of fulfillment and delivery of system policies (D_6_)	28	0.93	Y
Degree of administrative punishment at this level (including interviews, warnings, fines, etc.) (D_7_)	25	0.83	Y
Degree of completion of administrative reconsideration cases received within the period (D_8_)	16	0.53	N
Degree of completion of telephone/internet complaint cases within the stage (D_9_)	26	0.8	Y
Government authority (including regulation, information response, etc.) (D_10_)	27	0.9	Y
Image of the government (D_11_)	17	0.57	N
Reasonable degree of institutional setup (D_12_)	15	0.5	N
Administrative effectiveness (including departmental governance efficiency, degree of interdepartmental synergy, etc.) (D_13_)	26	0.86	Y
Level of pandemic literacy (D_14_)	15	0.5	N
Professional technology and risk assessment capacity (D_15_)	28	0.93	Y
Pandemic risk emergency response capacity (including research and evaluation capabilities, information dissemination channels, mobilization and decision-making, etc.) (D_16_)	30	1	Y
Ability to repair the relationship between the government and the people (communication ability, etc.) (D_17_)	27	0.9	Y
Degree of integrity and diligence of public officials (D_18_)	28	0.93	Y
Degree of pandemic control enforcement regulation (D_19_)	25	0.83	Y
Number of officials involved in corruption as a percentage of administrative personnel (D_20_)	14	0.47	N
Degree of accountability of public officials (D_21_)	16	0.53	N
Perceived procedural fairness (transparency, etc.) (D_22_)	25	0.83	Y
Perceived distributive justice (D_23_)	27	0.9	Y
Perceived interactional fairness (D_24_)	16	0.53	N
Sensitivity and responsiveness of public opinion (D_25_)	17	0.57	N
Public opinion collection and departmental transmission ability (D_26_)	27	0.9	Y
Ability to guide public opinion (D_27_)	29	0.97	Y
Ability to promote and educate pandemic governance theory (D_28_)	16	0.53	N
Physiological risk perception (D_29_)	29	0.97	Y
Property risk perception (D_30_)	17	0.57	N
Psychological risk perception (D_31_)	29	0.97	Y
Public satisfaction (D_32_)	30	1	Y
Level of political trust (D_33_)	27	0.9	Y
Level of political identification (D_34_)	15	0.5	N
Level of public participation in pandemic governance within the stage (D_35_)	15	0.5	N
Level of public participation in symposiums and hearings within the stage (D_36_)	25	0.83	Y
Degree of media access by people within the stage (the number of clicks on governmental platform visits, the number of likes, retweets, and comments on articles related to microblogs, Tik-Tok, and WeChat public numbers, etc.) (D_37_)	27	0.9	Y
Negative behaviors generated by people (smashing, blocking, fighting, rumor-mongering, etc.) (D_38_)	28	0.93	Y

Given that the public medical and health system costs are fuzzy concept, the public medical and health system costs indicator system are also considered to be a fuzzy set: {*Y*} was defined as the indicator set, i.e., each indicator in the indicator system was considered to be an element in the set, and then the expert survey method was used to conduct the indicator affiliation analysis and calculate the affiliation of 38 indicators of the public medical and health system costs. Based on this, we were able to determine whether to keep them in the public medical and health system costs index system. Assuming that the total number of expert confirmations for the *i*-th. indicator *Y*_*i*_ is *Z'*, i.e., a total of *Z*_*i*_ experts confirm *Y*_*i*_ as an important evaluation indicator for assessing the public medical and health system costs, and the total number of people measured is 30, then the affiliation degree of the public medical and health system costs indicator is:


(1) $$\begin{array}{l} R_{i}=\frac{Z_{i}}{30}\left(i=1,2,...,30\right) \end{array}$$

Whether the measurement index *Z*_*i*_ is taken or rejected depends on whether its membership, *R*_*i*_ is greater than or less than the critical membership. If it was the former, the measurement index was retained; if was the latter, it was deleted when critical membership = the critical value of expert selection times *Z/*30. Therefore, the critical value of expert confirmation times *Z* (α = 0.01) is:


(2) $$\begin{array}{l} Z=\mu +\frac{S}{\sqrt{Z^{\prime }}}t_{0.01}=23.4+\frac{5.77}{\sqrt{1140}}\times 2.368=23.8 \end{array}$$

In Formula 2, *S* denotes the standard deviation of the number of expert confirmations, μ denotes the expected value of the number of expert confirmations, *Z*′denotes the total number of expert confirmations, and *t*_0.01_ denotes the *T*-test value at a significance level of α = 0.01. An analysis of the data calculation showed that the critical affiliation was 79.4% when the critical value of the number of expert confirmations was *Z* = 23.8. Therefore, when the affiliation degree (α = 0.01) of an indicator was <79.4%, then the indicates was not statistically significantly different within the public medical and health system costs indicator system and was removed. Among the 38 indicators measuring the public medical and health system costs in the thesis, 13 indicators had an affiliation degree <79.4%, as detailed in Table 6(B). Thirteen indicators were removed from the 38 indicators in the second round of the indicator system construction process and 25 indicators were retained.

After expert testing and revision, this study finally determined the public medical and health system costs as a four-level index system with 40 indicators, of which the first-level indicator was the public medical and health system costs and the second-level indicator contained four points. Each secondary indicator had corresponding tertiary and quaternary indicators, which consisted of the original 10 tertiary indicators and 25 quaternary indicators identified by the redundancy analysis. In addition, the presentation of some of the level 4 indicators was corrected based on the results of the in-depth interviews (see Table 7).

**Table 7:** The public medical and health system costs indicator system after amendments.

**Primary indicators**	**Secondary indicators**	**Tertiary indicators**	**Quaternary indicators**
The public medical and health system costs	Institutional policy costs (B_1_)	Validity of institutional tools (C_1_)	Degree of effectiveness of policies and regulations enacted during the period (including regulation, governance measures, benefit compensation, etc.) (D_1_)
			Scientific basis and legitimacy of pandemic governance policy tools (including the degree of expert validation, assessment system, etc.) (D_2_)
			Completeness of the emergency prevention and control system (mechanism) (D_3_)
		Validity of system implementation (C_2_)	Consistency of system policy and government implementation (D_4_)
			Degree of fulfillment and delivery of system policies (including the implementation of norms) (D_5_)
			Degree of administrative punishment at this level (including interviews, warnings, fines, etc.) (D_6_)
			Degree of completion of telephone/internet complaint cases during the period (supervision, information response, etc.) (D_7_)
	System organizational costs (B_2_)	Government credibility and implementation (C_3_)	Government authority (including regulation, information response, etc.) (D_8_)
			Degree of interdepartmental synergy (D_9_)
		Competence level of public officials (C_4_)	Expertise and ability to assess risks (D_10_)
			Emergency response capacity of pandemic risks (research and evaluation capacity, information release channels, mobilization and decision making, etc.) (D_11_)
			Ability to repair the relationship between the government and the people (communication ability, etc.) (D_12_)
		Integrity level of public officials (C_5_)	Degree of integrity of public officials (D_13_)
			Degree of diligence of public officials (D_14_)
	Socially perceived costs (B_3_)	Perception of social equity (C_6_)	Perceived procedural fairness (transparency, etc.) (D_15_)
			Perceived fairness of distribution (D_16_)
		Effectiveness of public opinion regulation (C_7_)	Ability of public opinion collection and departmental transmission (D_17_)
			Ability to guide public opinion (D_18_)
		Social risk perception (C_8_)	Physiological risk perception (D_19_)
			Psychological risk perception (D_20_)
	Mass behavioral costs (B_4_)	Implicit participation behavior of the masses (C_9_)	Public satisfaction (D_21_)
			Political trust level (D_22_)
		Explicit participation behavior of the masses (C_10_)	Intensity of public participation in symposiums and hearings during the stage (D_23_)
			Degree of media access by people within the stage (the number of clicks on government platform visits, the number of likes, retweets and comments on articles related to microblogs, Shake, and WeChat public numbers, etc.) (D_24_)
			Negative behaviors generated by people (smashing, blocking, fighting, rumor-mongering, etc.) (D_25_)

### Confidence Validity Testing and Saturation Testing of the Coding

The Scott's *P*_*i*_ index was chosen as the reliability test when coding the nature of the public medical and health system costs. The formula for calculation is as follows:


(3) $$\begin{array}{l} Pi=\frac{X\%-Y\%}{1-Y\%} \end{array}$$

X = The number of analysis columns with identical coding results by two coders. Y = Expected value for consistency of coding results. The results are shown in Table 8.

**Table 8:** Degree of consistency of the coding.

**Category**	**Open**	**Axial**	**Selective**	**Total**
	**coding**	**coding**	**coding**	
Total number of codes	38	10	1	49
Number of consistency codes	31	10	1	42
X%	81.6	100	100	85.7

Scott's *P*_*i*_ was calculated to be 0.857 (generally, a value above 0.8 indicates good reliability), and therefore the coding part of the public medical and health system costs analysis was reliable. In order to ensure the validity of the coding and to further test the theoretical saturation of the public medical and health system costs measurement index system, the same or similar pandemic governance cases in China were selected for validation. In this paper, following the above standardized open coding procedures, three experts were selected to conduct saturation reliability analysis on the two groups of verification cases in the open coding process. Expert A analyzed a total of 176 original materials and codes in the two sets of cases, while Expert B and Expert C analyzed the same 100 source materials and codes for both sets of cases. One hundred source materials and codes analyzed by the three experts were taken as samples for reliability saturation analysis, and the results of the reliability saturation analysis are shown in Table 9.

**Table 9:** Results of coding saturation analysis.

**Category**	**A and B**	**B and C**	**A and C**
Number of codes that fully agree with each other	83	86	81
Average mutual agreement degree	0.83	0.86	0.81

The average agreement degree of the three was *K*≈0.833, and it was obtained that *R*≈0.85 through the reliability calculation formula, so the reliability of the coding was high. The saturation test results showed that: The categories in the index system had been edited and enriched, and no new categories or analogous relationships were found, which meant that the system had passed the coding validity test and reached saturation. Thus, the index system for the public medical and health system costs constructed in this study was scientific and reasonable.

## Application Analysis of the Measurement Index System

### Selection of Measurement Methods

The above analysis revealed that the public medical and health system costs index system was an abstract system and its measurement highlighted the problem of rational social choice and group decision making. The process of social choice and group decision making is based on how group members with common interests, different information, and different decision-making abilities join together to make the best decision (42). Whether it is through participation behavior or public activities, many different social choices and group decisions can play an extremely important role. Therefore, reasonable social choices and group decisions will directly affect the reduction in the public medical and health system costs consumption and the improvement in pandemic governance efficiency. In this kind of social choice and group decision process, the attribute values of the measured objects are mostly expressed in the form of fuzzy numbers or interval gray numbers. However, obtaining accurate and reasonable information about the true preferences and attributes of group decision making is often difficult because group utility functions are determined by well-defined individual utility functions (43), which means that assembling individual utility functions into group utility functions is a key aspect of the measurement process. The development of stochastic nonlinear utility and fuzzy decision theory has meant that most studies have tended to use fuzzy mathematical models to “synthesize” multiple evaluation index values into a holistic comprehensive evaluation value, and then obtain group preference relationships and group decision results. The attribute weights reflect the degree of importance that the assessor places on the assessment target, the degree of variation in the values of the assessment indicators, and the degree of reliability of the attribute values for the assessment target (44). Many methods have been used to obtain attribute weights, such as hierarchical analysis, the entropy method, etc. The multi-objective decision problems of social choice and group decision making can be transformed into single-objective decision problems through the calculation of weights (45). Attribute weights are also influenced by subjective and objective factors, and reasonable weight assignment methods are needed to improve the accuracy of weight assignment. The fuzzy comprehensive evaluation method refers to the use of multiple indicators based on a fuzzy set with hierarchical rows to make a comprehensive evaluation and classification of the subordinate rank status and change interval of the evaluated object. This method highlights the fuzzy nature of the evaluation criteria and influencing factors, and it can also combine qualitative and quantitative factors in the evaluation. This improves the accuracy and of the evaluation number, which means that evaluation conclusion is credible and thus it is more effective in evaluating complex problems with multiple factors and levels (46, 47). Furthermore, the public medical and health system costs are dynamic and comprehensive fuzzy concept, and are affiliation vector about multi-level rubrics where the rubric levels are grouped isometric ally.

Therefore, this study took the multi-attribute decision problem with a fuzzy number of attribute values as the starting point and used the G1 method to determine the subjective weight coefficients of each indicator of the public medical and health system costs. At the same time, the entropy weight method was used to determine the objective weight coefficients of each indicator of the public medical and health system costs. The level values of the public medical and health system costs of pandemic problems and the optimal combined G1/entropy weights of its indicators were determined by the combination method of subjective and objective assignments. Then, the descending semi-trapezoidal distribution method was selected based on the evaluation level of each indicator, the affiliation distribution function was calculated, and the fuzzy transformation principle was used to determine the value of the public medical and health system costs. This methodology meant that it was possible to clearly and reliably conclude the group decision made by some individuals and judge the real attitudes and choices of actors about pandemic problem coping strategies. This meant that the measurement of the public medical and health system costs had wider non-contingency, applicability and practicality.

### Measurement Design and Data Acquisition

The questionnaire about the public medical and health system costs are designed around the understanding ability of villagers and citizens based on the 25 four-level indicators of the index. The questionnaire contained nine items of basic information about the respondents and 25 items that were scored on a seven-point Likert scale. The target of the scale was still the pandemic prevention and control event in S City, and it was gradually improved by consulting experts from universities and research institutes in the Beijing-Tianjin-Hebei region, local people, surrounding village cadres, and government authorities, etc. The objective weights of the four levels of indicators were calculated from the questionnaire and the public medical and health system costs to the government of S City when managing the pandemic were measured. This study questionnaire survey was conducted in two phases according to the development of events, and was carried out in parallel with interviews and resident research. The survey was conducted using the random distribution method and face-to-face interviews. The survey and processing of the first phase of the questionnaire took place between December 25, 2020 and January 11, 2021. The second phase of the survey and processing of the questionnaire took place between December 1, 2021 and December 21, 2021. A total of 400 questionnaires were distributed to meet the sampling requirements, of which 377 questionnaires were collected, with a recovery rate of 94.2%. There were 309 valid questionnaires, which was an effective rate of 82%.

Given the duplication of measurement processes in the two questionnaire phases, the first phase of the research questionnaire and data were selected to analyze the process of assigning and measuring the public medical and health system costs. Prior to each phase of the formal survey, a pretest was conducted to ask whether the subjects could clearly understand and easily respond. Then, the questionnaire was revised in several rounds until it showed good internal validity. The Cronbach's α values for the four measurements of the public medical and health system costs of the questionnaire study were 0.728, 0.726, 0.714, and 0.702, which showed that the measurements had excellent reliability levels. The scale was purified using the Corrected Item-Total Correlation (CITC). Question items with a CITC value of 0.5 or below were considered less relevant to the overall results. If Cronbach's α increased significantly when these question items were deleted, it was decided that these questions should be deleted to achieve scale purification. The results showed that all 25 question items passed the test, that is, the Cronbach's α coefficient of each indicator was greater than the critical value of 0.6, which meant they were highly reliable and there was no need to delete any question items. The validity test of the questionnaire used in the first questionnaire phase analysis used the Kaiser-Meyer-Olkin (KMO) measurement and Bartlett's sphericity test. After testing, the KMO values of four of the indicators were >0.6, and thus the questionnaire had significant structural validity. The Bartlett's test of significance was 0.000, and the significance expression met the requirements, so the questionnaire had high validity and could be used for objective weighting and the measurement of the public medical and health system costs.

### Combination of Subjective and Objective Empowerment and Fuzzy Measurements

#### G1 Method to Determine the Weights of the Measurement Indicators

The G1 method improves on the AHP (Analytic Hierarchy Process) method by getting rid of the constraint that decision makers must construct a judgment matrix when judging the scheme, avoids the disadvantage that it is difficult to meet the consistency requirements of the AHP method, and fundamentally solves the consistency problem of individual judgment. Furthermore, the G1 method does not need a consistency test. This method is especially suitable for weighting large scale index systems with many factors. Therefore, the G1 method was selected for the subjective weighting.

In this study, seven experts who have been engaged in the research fields of pandemic governance and public crisis management for many years were invited. After judging the relationship of each indicator to the public medical and health system costs, their importance was assigned as follows:

Let *x*_1_*, x*_2_*, x*_3_*, …, x*_*m*_ *(m* ≥ 2*)* be *m* extremely large indicators that have undergone uniformization and non-dimensionalization of indicator types.

Definition 1: If the importance of index *x*_*i*_ relative to a certain evaluation criterion (or target) is not inferior to *x*_*j*_, it is recorded as *x*_*i*_ ≥*x*_*j*_ (the symbol ≥ indicates that it is not an inferior relationship).

Definition 2: If index *x*_1_*, x*_2_*, x*_3_*, …, x*_*m*_ has a relationship with an evaluation criterion (or objective):


(4) $$\begin{array}{l} x_{i}\geq x_{j}\geq \cdots \;x_{k}\left(i,j,\ldots ,k=1,2,\ldots m\right) \end{array}$$

then it is said that the evaluation indexes *x*_1_*, x*_2_*, x*_3_*, …, x*_*m*_ have established an order relationship according to “≥”.

For the evaluation index set {*x*_1_*, x*_2_*, x*_3_*, …, x*_*m*_}, the order relationship can be established according to the following steps:

Select the most important or least important of the *m* indicators and mark it as *x*_*i*_;

Select the most important or least important indicator among the remaining *m-1* indicators and mark it as *x*_*j*_;

Select the most important or least important indicator among the remaining *m-(k-1)* indicators and mark it as *x*_*n*_; Mark the remaining indicator as *x*_*k*_.

After determining the only order relationship, the next step is to determine the importance of adjacent indicators.

Under the selected scale, the rational judgment of the selected experts on the importance ratio *w*_*k*−1_*/w*_*k*_ between adjacent indexes *x*_*k*−1_ and *x*_*k*_ is derived as follows:


(5) $$\begin{array}{l} w_{k-1}/w_{k}=r_{k}\left(k=m,m-1,m-2,\ldots ,3,2\right) \end{array}$$

When the number of indicators is large, the most minor indicator *r*_*m*_ = 1 can be used and the *r*_*k*_ value can be derived from Table 10.

**Table 10:** The scale.

***r_***k***_***	**Definition**
1.0	Indicator *x_*k*−1_* is as important as indicator *x_*k*_*
1.2	Indicator *x_*k*−1_* is slightly more important than indicator *x_*k*_*
1.4	Indicator *x_*k*−1_* is obviously more important than indicator *x_*k*_*
1.6	Indicator *x_*k*−1_* is more important than indicator *x_*k*_*
1.8	Indicator *x_*k*−1_* is extremely more important than indicator *x_*k*_*

The information and data filled in by the experts can then be combined to calculate the indicator weights layer by layer using the G1 method.

If the expert gives a rational assignment of *r*_*k*_, the weight $w_{j}^{g}$ is:


(6) $$\begin{array}{l} w_{j}^{g}={\left(1+\overset{m}{\underset{k=2}{\sum }}\underset{i=k}{\overset{m}{\prod }}r_{i}\right)}^{-1} \end{array}$$

(7) $$\begin{array}{l} w_{k-1}=r_{k}w_{k}\left(k=m,m-1,m-2,\ldots ,3,2\right) \end{array}$$

Finally, the single-tier subjective weights and comprehensive weights of 1 primary indicator, 4 secondary indicators, 10 tertiary indicators and 25 quaternary indicators were completed after collating the assignment results, as shown in Table 11.

**Table 11:** Combined weights of the indicators for measuring the public medical and health system costs in the first phase of the questionnaire process.

**Primary Indicator**	**Secondary Indicator Single-tier weighting**	**Tertiary indicators Single-tier weighting**	**Quaternary indicators Single-tier weighting**	**Composite** **Subjective weighting** **$W_{g}^{j}$**	**Questionnaire Objective weighting $W_{s}^{j}$**	**Portfolio Weighting $W_{z}^{j}$**
(A)1	(B_1_) 0.2362	(C_1_) 0.4167	(D_1_) 0.4248	0.0418	0.0409	0.0414
			(D_2_) 0.3539	0.0348	0.0400	0.0374
			(D_3_) 0.2213	0.0218	0.0382	0.0300
		(C_2_) 0.5833	(D_4_) 0.2049	0.0282	0.0351	0.0317
			(D_5_) 0.3443	0.0474	0.0350	0.0412
			(D_6_) 0.2459	0.0339	0.0417	0.0378
			(D_7_) 0.2049	0.0282	0.0359	0.0321
	(B_2_) 0.2362	(C_3_) 0.2778	(D_8_) 0.3846	0.0252	0.0408	0.0330
			(D_9_) 0.6154	0.0404	0.0382	0.0393
		(C_4_) 0.4444	(D_10_) 0.2212	0.0232	0.0396	0.0314
			(D_11_) 0.4248	0.0446	0.0376	0.0411
			(D_12_) 0.3540	0.0372	0.0387	0.0379
		(C_5_) 0.2778	(D_13_) 0.5000	0.0328	0.0395	0.0362
			(D_14_) 0.5000	0.0328	0.0416	0.0372
	(B_3_) 0.1969	(C_6_) 0.3017	(D_15_) 0.4167	0.0248	0.0448	0.0348
			(D_16_) 0.5833	0.0347	0.0403	0.0375
		(C_7_) 0.2155	(D_17_) 0.6429	0.0273	0.0407	0.0340
			(D_18_) 0.3571	0.0152	0.0383	0.0267
		(C_8_) 0.4828	(D_19_) 0.6154	0.0585	0.0387	0.0486
			(D_20_) 0.3846	0.0366	0.0368	0.0367
	(B_4_) 0.3307	(C_9_) 0.4167	(D_21_) 0.5455	0.0752	0.0391	0.0571
			(D_22_) 0.4545	0.0626	0.0366	0.0496
		(C_10_) 0.5833	(D_23_) 0.2033	0.0392	0.0416	0.0404
			(D_24_) 0.2846	0.0549	0.0494	0.0521
			(D_25_) 0.5121	0.0988	0.0508	0.0748

*The calculation results are retained to four decimal places*.

#### Determination of the Weights of the Measurement Indicators by the Entropy Method

The importance of the 25 question items is calculated in strict accordance with the steps of the entropy value method for assigning weights, and the objective weights of the public medical and health system costs (four levels of indicators) were derived. Since the objective weighting of the public medical and health system costs measurement index used the questionnaire survey data, there was no need for dimensionless processing of the data, and the calculation formula is as follows.

Index information entropy:


(8) $$\begin{array}{l} e_{j}=-k\overset{n}{\underset{i=1}{\sum }}Y_{ij}^{\prime }\ln\;Y_{ij}^{\prime },j=1,2,3\ldots ,m \end{array}$$

Index redundancy:


(9) $$\begin{array}{l} d_{j}=1-e_{j},j=1,2,3,\ldots ,m \end{array}$$

Index entropy:


(10) $$\begin{array}{l} w_{j}^{s}=d_{j}/\overset{m}{\underset{j=1}{\sum }}d_{j},j=1,2,3,\ldots ,m \end{array}$$

where: *e*_*j*_ is the information entropy $Y_{ij}^{\prime }=Y_{ij}/\sum_{i=1}^{n}Y_{ij}$ of index *j*; *d*_*j*_ is the redundancy of index *j*; $w_{j}^{s}$ is the entropy weight of index *j*; *m* is the number of indicators, *n* is the number of questionnaires, and *k* is the adjustment coefficient (*k* = 1/ln *n*).

The calculation results are shown in Table 11.

#### Portfolio Weights

The key problem of combined weighting is how to determine the weight distributions of the two methods. An extensive literature review found that most researchers had used the method of subjective and objective average weighting with the minimum sum of squared deviations as the objective function to calculate the combined weight. Furthermore, this study calculated the public medical and health system costs on the basis of the identified indicator system, without the need for indicator rejection and without the need to reflect the differences between expert knowledge and experience, and the objective data. The results suggested that the multiplicative synthetic normalization method with the linear weighted combination method was suitable for calculating the combined weighting.

Suppose $w_{j}^{z}$ is the *j*-th index weight obtained by a linear combination of the two weighting methods, that is:


(11) $$\begin{array}{l} w_{j}^{z}=\lambda w_{j}^{g}+\left(1-\lambda \right)w_{j}^{s} \end{array}$$

where λ is the proportion of the index weight determined by the G1 method in the combined weight. The objective function is constructed by minimizing the deviation between the index weight determined by the G1 method and the combined weight, and the square sum of the deviation between the index weight determined by entropy method and the combined weight is:


(12) $$\begin{array}{l} \text{min }z=\overset{n}{\underset{j=1}{\sum }}\left[{\left(w_{j}^{z}-w_{j}^{g}\right)}^{2}+{\left(w_{j}^{z}-w_{j}^{s}\right)}^{2}\right] \end{array}$$

From Formula (12), we get:


(13) $$\begin{array}{l} \text{min }z=\overset{n}{\underset{j=1}{\sum }}{\left[\lambda w_{j}^{g}+\left(1-\lambda \right)w_{j}^{s}-w_{j}^{g}\right]}^{2}\\ \;+{\left[\lambda w_{j}^{g}+\left(1-\lambda \right)w_{j}^{s}-w_{j}^{s}\right]}^{2} \end{array}$$

Then, find the derivative of Formula (13) with respect to λ and set the first-order derivative to 0 to obtain λ = 0.5. Substituting λ = 0.5 into Formula (11) allows, the weight value after the combined weighting to be can be obtained as follows:


(14) $$\begin{array}{l} w_{j}^{z}=0.5w_{j}^{g}+0.5w_{j}^{s} \end{array}$$

The subjective and objective weights of the indicators for measuring the public medical and health system costs are unified by combining the objective weights obtained by the entropy method with the subjective comprehensive weights for each indicator obtained by the G1 method according to the above formula. These weights can then be used to calculate the combined weight of each indicator. The calculation results are shown in Table 11.

The overall weight rankings of the indicators for the public medical and health system costs in Table 11 show that although the G1 method and entropy method had different emphases in the calculation and analysis of indicator weights, there was still a high degree of consistency in the subjective ranking, objective ranking, and overall ranking results for more than half of the indicators. This result also indicates, to a certain extent, that the combined weighting method based on the G1 method and entropy method was scientific and applicable.

#### Fuzzy Integrated Measurement

Step 1. After calculating the combined weights of the public medical and health system costs indicators, the final measurement analysis was carried out according to the above fuzzy comprehensive evaluation method. The data were chosen from the questionnaire data for the area surrounding the studied case in the first phase of the questionnaire process.

Establish the set of evaluation factors U. Public medical and health system costs: U = {U1, U2, U3, U4}. Institutional policy costs: U1 = {U11, U12}. System organizational costs: U2 = {U21, U22, U23}. Socially perceived costs: U3 = {U31, U32, U33}. Mass behavioral costs: U4 = {U41, U42}. Validity of institutional tools: U11 = {U111, U112, U113}. Validity of system implementation: U12 = {U121, U122, U123, U124}. Government credibility and implementation: U21 = {U211, U212}. Competence level of public officials: U22 = {U221, U222, U223}. Integrity level of public officials: U23 = {U231, U232}. Perceived social equity: U31 = {U311, U312}. Effectiveness of public opinion regulation: U32 = {U321, U322}. Social risk perception: U33 = {U331, U332}. Implicit participation behavior of the masses: U41 = {U411, U412}. Explicit participation behavior of the masses: U42 = {U421, U422, U423}.

Step 2. The set of comments (V) were determined where V= {strongly agree, agree, relatively agree, average, relatively disagree, disagree, strongly disagree} = {7, 6, 5, 4, 3, 2, 1} respectively.

Step 3. The factor weights and sub-factor weights were then determined. Here the weight values (values were calculated to three decimal places) after the combination assignment in the previous subsection were used.

A = (0.285, 0.256, 0.218, 0.274). A1 = (0.109, 0.143). A2 = (0.072, 0.110, 0.073). A3 = (0.072, 0.061, 0.085). A4 = (0.107, 0.167). A11 = (0.041, 0.037, 0.030). A12 = (0.032, 0.041, 0.038, 0.032). A21 = (0.033, 0.039). A22 = (0.031, 0.041, 0.038). A23 = (0.036, 0.037). A31 = (0.035, 0.037). A32 = (0.034, 0.027). A33 = (0.049, 0.037). A41 = (0.057, 0.050). A42 = (0.040, 0.052, 0.075).

Step 4. The fuzzy comprehensive evaluation matrix calculation for the sub-factor set was performed, where:


$$\begin{array}{l} R_{11}\text{=}\left\{\begin{array}{c} R_{111}\\ R_{112}\\ R_{113} \end{array}\right\}=\left\{\begin{array}{c} r_{1111}...r_{1117}\\ r_{1121}...r_{1127}\\ r_{1131}...r_{1137} \end{array}\right\} \end{array}$$

……


(15) $$\begin{array}{l} R_{42}\text{=}\left\{\begin{array}{c} R_{421}\\ R_{422}\\ R_{423} \end{array}\right\}=\left\{\begin{array}{c} r_{4211}...r_{4217}\\ r_{4221}...r_{4227}\\ r_{4231}...r_{4237} \end{array}\right\} \end{array}$$

$$\begin{array}{l} B_{11}=A_{11}▪R_{11} \end{array}$$

……


(16) $$\begin{array}{l} B_{42}=A_{42}▪R_{42} \end{array}$$

(17) $$\begin{array}{l} B=B_{11}+...+B_{42} \end{array}$$

Step 5. The fuzzy comprehensive evaluation result vector was synthesized. The fuzzy comprehensive evaluation result vector was obtained after inputting the measurement results for all the public medical and health system costs indicators into the judgment matrix as follows:


(18) $$\begin{array}{l} B=B_{11}+...+B_{42}\\ \;=\left(0.050,0.126,0.204,0.296,0.156,0.123,0.044\right) \end{array}$$

According to the principle of maximum affiliation, the evaluation set for the public medical and health system costs indicators was considered to be “average,” and the feature vector was 0.296.

Step 6. A comprehensive measurement analysis of the result vector for the fuzzy comprehensive evaluation was undertaken. The comprehensive score for the public medical and health system costs measurements was obtained from θ = *B* × *D*^*T*^ = 4.071. That is, in the pandemic governance at the S City, the measured value for the public medical and health system costs produced by the first stage of the pandemic governance was 4.071.

Given the different attributes and magnitudes of the public medical and health system costs indicators in the two governance stages, the objective questionnaires used to measure the two stages were different. Furthermore, the resulting objective factor weights and fuzzy evaluation composite values were also different. Although the assignment of experts allows for comprehensive judgment and subjectivity, and the same weight is given to both stages, the combination of the objective questionnaire data conducted simultaneously with the two governance stages still resulted in a more reasonable and accurate calculation of the public medical and health system costs indicator weights for each stage. The above calculation process gives a value of 4.133 for the public medical and health system costs indicators during the second governance stage in S City. In the survey, the scoring items in the seven-point Likert scale were used and were scored based on the respondents' agreement with the question items in descending order. When the public medical and health system costs measurement was <4.071, the public medical and health system costs and benefit to the pandemic governance are in a balanced state, and the local pandemic governance is in a safe and stable period. However, when the public medical and health system costs measurement is >4.071, then the public medical and health system costs are viciously consumed and the local pandemic governance system has entered a dangerous period. The government needs to focus on the public medical and health system costs.

## Conclusions and Policy Implications

### Main Conclusions

After measuring the combined weights of the public medical and health system costs, it can be concluded that the weights of the measured indicators evolved according to the different temporal and spatial states of the measured objects and pandemic governance events, and the importance of each indicator in each stage to the public medical and health system costs varied. Among them, mass behavioral costs and the five categories that included fourth-level indicators had the greatest weight among all the indicators and had a greater impact on the public medical and health system costs results. They are also the core elements affecting the public medical and health system costs consumption. And three other the public medical and health system costs (institutional policy, system organizational, and social perception) that have a more balanced degree of influence. In the four-level indicator system, the weight of “negative behaviors generated by people” is the largest among the stages, and “public satisfaction” is the second largest factor affecting the public medical and health system costs, so the government must focus on the analysis public interest of pandemic governance. The other four levels of indicators significantly fluctuated in their weights at each stage depending on the nature of the event. In addition, when the reliability and validity of the questionnaire were tested, it was found that the scores for respondent evaluation of institutional policy and system organizational costs caused to pandemic governance were more consistent, and the values of these two indicators in the questionnaire were high in the reliability and validity tests. The variability of the respondent evaluation scores on the social perception costs of pandemic problems and the behavioral cost of the masses indicators were greater than for the other indicators, which makes the values of these two indicators in the questionnaire low in the reliability and validity tests. These two situations are consistent with the assumptions of the pre-survey and the actual situation highlighted by the field research.

The public medical and health system costs of the pandemic governance in S City were already evident during the first stage of the COVID-19 pandemic governance event. However, because the local government did not pay much attention to it due to reasons such as “employment rates,” “local stability and security” and “increase in local GDP,” the public medical and health system costs evolved during the first stage. In the first stage, local governments failed to provide timely guidance, treatment, and systems to address the problems and public opinion information reflected by the public. At the same time, the public medical and health system costs in the first stage was 4.071, which meant that The public medical and health system costs significantly increased during this stage and was at the critical point of the “cost-benefit” equilibrium for the government to manage pandemic problems. In this context, if the local government does not pay attention to the problem, it will inevitably evolve into the second stage. The second stage witnessed negative public behavior, and negative public psychology began to expand and spread rapidly. However, the failure of local government to carry out effective public emergency responses and governance led to the continuous expansion of the public medical and health system costs consumption. At this point, the measured value for The public medical and health system cost was 4.133, suggesting that the S City government should introduce and adopt timely governance policies and invest more active governance efforts to control the continued rise in the public medical and health system costs depletion. This index system shows that the measurement results have high consistency with the actual event evolution results, and it also proves that the measurement index system and measurement method for ascertaining the public medical and health system costs constructed in this study are practical. Therefore, when practicing local pandemic governance, the government can use the index system and measurement method to measure the size of the public medical and health system costs in the region and use it as an important basis for measuring the performance of pandemic governance.

The risk society theory is combined with the “cost-utility” theory in this paper. In line with the case analysis and cost measurement, it is concluded that local governments often take advantage of system resources for rent-seeking and result in high system costs in the process of pandemic prevention and control. Coupled with the system costs inflation caused by the wrong decision-making of some local governments and the underestimate of virus risk, the “cost-benefit” imbalance in the supply of public goods in the process of pandemic prevention and control is caused. The local governments as the “Rational Economic Man” that take the pursuit of maximizing their own interests as their own behavioral motivation. After that, the best scheme is selected by them to realize their own interests. In this case, the weaker collinearity between the regional pandemic control benefits and the overall level of local governance is presented. Since the externalities of the pandemic control are shown, the public interest is usually composed of political, social, economic and other multidimensional elements, the positive externalities are far from enough for local governments to guarantee both economic efficiency and social equity. The local governments will deviate from the public's agency goals of pandemic control to a certain extent due to the lack of reasonable constraints and supervision, so the certain risks will be caused. In view of it, the transmission nature and critical connection between cost-utility theory and risk society theory are proved in this thesis. Therefore, the asymmetry in the allocation of their own interests and the interests of other subjects such as the public should be focused on by the local governments, the payment cost of the client in the pandemic control should be paid attention to, the public choice theory should be introduced. In short, a perfect cost-benefit indicator management system and an efficient citizen participation mechanism should be set up, the system should be rationally formulated, and the participation channels of other governance subjects should be broadened, so various “transaction costs” in the process of pandemic governance can be reduced. In this thesis, the crucial role of the public choice theory in pandemic prevention and control is demonstrated. The costs of public health and medical system are taken as one of the variables to evaluate the efficiency of local government pandemic governance, so the research on pandemic governance will be more systematic and pertinent.

### Policy Implications

Local governments should establish the public medical and health system costs awareness and adopt appropriate assessment and enforcement efforts. And local governments should establish a the public medical and health system costs control mechanism and an automatic identification and monitoring mechanism, make good use of the emotional buffer in the virtual space of the network, and pay attention to early warnings during each stage of pandemic governance in order to issue and adopt governance policies in a timely manner to control the consumption of the public medical and health system costs. At the same time, local governments often form their own preferences based on local economic benefits, taxation, and governance costs to determine the intensity of pandemic governance policies. When economic benefits are consistent with local pandemic governance preferences, local governments are prompted to implement governance policies while ignoring the public medical and health system costs. When the two preferences are inconsistent, local governments often adopt incomplete implementation and avoidance of responsibility for scientific pandemic governance. However, the public medical and health system resources held by governance active subjects when managing the pandemic are limited. Therefore, this approach will increase the consumption of the public medical and health system costs, will have an impact on social stability, the business environment, and later policy implementation, and will also hinder local economic development. Therefore, it is important that public officials improve their professionalism. They should be equipped with professional instruments and technical facilities to improve the collection and analysis of pandemic governance monitoring, to enable them to make rapid responses and decisions to some problems in pandemic governance, and to pre-design, accurately predict, and “advance” governance. It is also necessary to appropriately control the administrative accountability of officials, gradually increase public reputation evaluation, carefully evaluate whether the policy objectives and the public medical and health system costs match, and reasonably divide the boundary between social supervision and government supervision to prevent policy overflow and policy overrun.

Internalize the externalities of pandemic governance and reduce the cost of public participation. Public behavior is a core element that affects the consumption of the public medical and health system costs. When medical system resources for pandemic governance are depleted, the public will give up consideration of economic interests, health, and other factors, and choose a behavioral strategy of negative participation and collective protest, so that the governance system becomes unstable. This is not only a warning to remind some public officials who follow the risky behavior of concealing information asymmetry, but will also give the heads of government reassurance. When it comes to pandemic governance issues, officials should “let go” so that the governance of pandemic issues can be more effectively implemented. At the same time, further promoting government efforts to strengthen its active governance strategy may have a negative impact on local economic development (reduce the rent and lower taxation), but it is possible to seek appropriate feedback paths for this, such as transfer payment funds could be used to reduce their overall tax burden, build a complete pandemic monitoring system and monitoring information system, or provide corporate subsidies for technical innovation improvement total factor productivity-economic growth-drives local governments to increase investment in pandemic governance and is a benign interactive cycle chain. In addition, the rational perception of the public toward pandemic governance should be improved through various channels and platforms, such as official media and social organizations, and the public‘s psychological intervention mechanism should be improved. It is also possible to carry out cross-departmental and cross-organization collaborative governance, determine the subsidy intensity based on the public perception of pandemic governance, efficiently handle public letters, visits, complaints and other communication, rationally design participation forms, broaden public participation channels, and reduce public participation costs. This will help to correct public bias and beliefs, promote value recognition, improve public psychological morale.

### Limitation and Future Direction

The increasing demands to construct an indicator system and the associated scientific and rigorous selection of indicators mean that it is necessary to consider both micro and macro effects when constructing the indicator system, and to pay attention to both current and long-term interests, i.e., to be comprehensive and focused. Therefore, this study adopted a scientific, statistical method that combined qualitative and quantitative analyses to construct an indicator system for the public medical and health system costs that passed the relevant tests. It also underwent improvements, resulting in a more reasonable structure for the indicator system, a more appropriate number of indicators, and good reliability and validity. This method is a stable and effective measurement framework, which lays a foundation for future research and fills part of the research gap in the public medical and health system costs. It is hoped that this study will help local governments identify the main factors affecting the changes in the public medical and health system costs so that they can propose governance solutions and strategies from a multidimensional perspective, and build a common and integrated public policy support system by 2050 for the emergency management organization system to help with the prevention and control of major pandemics, with joint guarantees for restoration and a stronger post-pandemic collaborative governance network, so as to restore infrastructure, social property, social order, public morale, and government credibility to normal as soon as possible, and precisely control the public medical and health system costs, and better improve the quality and efficiency of pandemic governance. The study of the public medical and health system costs is inevitably a long-term and complex systematic subject. Since there are relatively few related studies in China, and the theory and research system are not perfect, this study had some shortcomings. For example, the concept and index system for the public medical and health system costs may need to be further expanded, and the measurement method should be improved and upgraded, both of which will be addressed in future studies. At the same time, this study will serve as a beginning and contribute to the study of the public medical and health system costs.

## Data Availability Statement

The original contributions presented in the study are included in the article/Supplementary Material, further inquiries can be directed to the corresponding author/s.

## Ethics Statement

The studies involving human participants were reviewed and approved by Biomedical Ethics Committee of Nankai University No. NKUIRB2022092. The patients/participants provided their written informed consent to participate in this study.

## Author Contributions

XL was responsible for reviewing and editing, conceptualization, visualization, software, and methodology. ZZ was responsible for validation and investigation. LL was responsible for supervision. TC was responsible for validation. GL was responsible for visualization. All authors contributed equally to this work, read, and approved the final manuscript.

## Funding

This study was supported by the Tianjin Social Science Foundation of China under Grant no. TJGLQN20-001, China Postdoctoral Science Foundation under Grant no. 2020M670636, and Fundamental Research Funds for the Central Universities of China under Grant no. 63212079. The funders had no role in the study design, data collection and analyses, the decision to publish, and the preparation of the manuscript.

## Conflict of Interest

The authors declare that the research was conducted in the absence of any commercial or financial relationships that could be construed as a potential conflict of interest.

## Publisher's Note

All claims expressed in this article are solely those of the authors and do not necessarily represent those of their affiliated organizations, or those of the publisher, the editors and the reviewers. Any product that may be evaluated in this article, or claim that may be made by its manufacturer, is not guaranteed or endorsed by the publisher.

## Footnotes

^1^A gatekeeper is a person who has authority over the researched person. The gatekeepers include the head of the pandemic governance unit and government. When arriving in S City in early November, 2020 to try to understand the situation from villagers, we were interfered with by the village committee, and the town government's general government office. It was only after three days and an introduction letter from the Center for Disease Control and Prevention in S City that the investigation could be launched.
