# Supplementary material for: Analysis of the Quantitative Evaluation of the Public Medical and Health System Costs During Pandemic Governance: Investigation Based on COVID-19
Source: Front Public Health. 2022 Jul 13;10:942043. doi: 10.3389/fpubh.2022.942043 (PMC9326215; doi:10.3389/fpubh.2022.942043)
Supplement: Supplementary file 2 [file Table_2.DOC]

**公共医疗卫生制度成本测度的调查问卷**

尊敬的女士／先生：

您好!

近些年由于新冠疫情一定程度上影响到您的生活、工作等方面。本调查是天津市社科基金项目“重大疫情防控中应急管理组织体系的协同效应及优化对策研究”的问卷部分，想了解一下您对公共医疗卫生制度的真实想法，以便我们从成本角度推进疫情治理政策措施的完善和优化。根据《中华人民共和国统计法》，所有的调查资料仅为学术研究之用，您的回答将对外绝对保密。您的见解和意见对本课题的完成至关重要，填写问卷会占用您一些宝贵时间，我们衷心感谢您的支持和参与！

“天津市卫生应急能力提升质量控制中心”课题组

填写说明：本问卷中的选择题均为单选题，请在符合您实际情况的选项或相应的数字上打（√）或者在“_____”上填写您回答的内容（对题项认同程度：7=非常认同；6=认同；5=比较认同；4=一般；3=比较不认同；2=不认同；1=非常不认同）。问卷调查所涉及的问题没有对错之分，您只需要按照实际情况填写，您答案的真实性和完整性对本课题的质量有重要影响。

一、问卷填写

1.制度性成本调查

| **题项** | **非常认同** | **认同** | **比较认同** | **一般** | **比较不认同** | **不认同** | **非常不认同** |
| --- | --- | --- | --- | --- | --- | --- | --- |
| **地方政府在防控疫情时使用的政策法规都有效果** | **7** | **6** | **5** | **4** | **3** | **2** | **1** |
| **地方政府在疫情防控过程中采取的方式行为都合理科学（经过科学论证评估并有合法渠道）** | **7** | **6** | **5** | **4** | **3** | **2** | **1** |
| **地方政府在疫情防控过程中具有明确清晰的应急管理制度** | **7** | **6** | **5** | **4** | **3** | **2** | **1** |
| **地方政府在疫情防控过程中政策执行与政策内容一致** | **7** | **6** | **5** | **4** | **3** | **2** | **1** |
| **地方政府在疫情防控时都履行和兑现了政策中要求的承诺** | **7** | **6** | **5** | **4** | **3** | **2** | **1** |
| **地方政府在疫情防控时对所属或者下级部门、企业以及相关人员治理措施明确** | **7** | **6** | **5** | **4** | **3** | **2** | **1** |
| **地方政府在疫情防控过程中对电话/网络投诉案件都及时办结** | **7** | **6** | **5** | **4** | **3** | **2** | **1** |

2.组织性成本调查

| **题项** | **非常认同** | **认同** | **比较认同** | **一般** | **比较不认同** | **不认同** | **非常不认同** |
| --- | --- | --- | --- | --- | --- | --- | --- |
| **地方政府在疫情防控时能政令畅通，令行禁止，监管到位** | **7** | **6** | **5** | **4** | **3** | **2** | **1** |
| **地方政府在疫情防控时各部门办事效率高、部门配合程度好** | **7** | **6** | **5** | **4** | **3** | **2** | **1** |
| **地方政府等工作人员在疫情防控过程中具有很高的专业技术水平** | **7** | **6** | **5** | **4** | **3** | **2** | **1** |
| **地方政府等工作人员在疫情防控、面对风险时的应急管理能力很高** | **7** | **6** | **5** | **4** | **3** | **2** | **1** |
| **地方政府等工作人员在疫情防控时与我们的关系一直处理融洽** | **7** | **6** | **5** | **4** | **3** | **2** | **1** |
| **地方政府等工作人员在疫情防控过程中很勤政，很积极有作为** | **7** | **6** | **5** | **4** | **3** | **2** | **1** |
| **地方政府等工作人员在环境治理执法都很规范** | **7** | **6** | **5** | **4** | **3** | **2** | **1** |

3.社会感知性成本调查

| **题项** | **非常认同** | **认同** | **比较认同** | **一般** | **比较不认同** | **不认同** | **非常不认同** |
| --- | --- | --- | --- | --- | --- | --- | --- |
| **地方政府在疫情防控时出台和执行政策都有公平公开的程序** | **7** | **6** | **5** | **4** | **3** | **2** | **1** |
| **地方政府在疫情防控时出台和执行政策都会使我们受益** | **7** | **6** | **5** | **4** | **3** | **2** | **1** |
| **地方政府在疫情防控过程中对污染事件的信息以及我们的意见能够及时收集，并能做有效布置** | **7** | **6** | **5** | **4** | **3** | **2** | **1** |
| **地方政府在疫情防控时对任何事件都会准确及时向我们报道，并安抚和引导我们情绪** | **7** | **6** | **5** | **4** | **3** | **2** | **1** |
| **地方存在的疫情问题没有影响到我的身体健康** | **7** | **6** | **5** | **4** | **3** | **2** | **1** |
| **地方存在的疫情问题没有让我产生不好的心理情绪** | **7** | **6** | **5** | **4** | **3** | **2** | **1** |

4.行为性成本调查

| **题项** | **非常认同** | **认同** | **比较认同** | **一般** | **比较不认同** | **不认同** | **非常不认同** |
| --- | --- | --- | --- | --- | --- | --- | --- |
| **我对地方政府在疫情防控过程中的政策以及都做法很满意** | **7** | **6** | **5** | **4** | **3** | **2** | **1** |
| **我对地方政府在疫情防控过程中的政策以及做法都很支持和信任** | **7** | **6** | **5** | **4** | **3** | **2** | **1** |
| **我都参与了地方政府举办的有关疫情治理方面的座谈会、听证会等** | **7** | **6** | **5** | **4** | **3** | **2** | **1** |
| **我一直在关注政府网站、微博、微信、电视等媒体中对地方周边疫情问题的报道** | **7** | **6** | **5** | **4** | **3** | **2** | **1** |
| **一旦地方疫情严重，我不会与其他同伴参加示威和其他抗议活动** | **7** | **6** | **5** | **4** | **3** | **2** | **1** |

二、人口统计学特征

1、您的性别：

（1）男□ （2）女□

2、您的年龄是：

（1）18-30岁□（2）31-40岁□ （3）41-50岁□ （4）51-60岁□

（5）60岁以上□

3、您的户籍类型：

（1）城镇□ （2）农村□

4、您在此地居住时间：

（1）10年以下□ （2）11-20年□ （3）21-30年□ （4）31-40年□

（5）40年以上□

5、您对本地政府在防控疫情的宣传是否满意：

（1）非常满意□ （2）满意□ （3）比较满意□ （4）一般□

（5）比较不满意□

（6）不满意□ （7）非常不满意□

6、在本地稍缓后，您是否愿意继续住在本地区：

（1）非常愿意□ （2）愿意□ （3）比较愿意□ （4）一般□

（5）比较不愿意□ （6）不愿意□ （7）非常不愿意□

7、您的文化程度：

（1）小学及以下□ （2）中学学历□ （3）大学学历□ （4）研究生及以上□

8、您的工作单位是：

（1）机关单位□ （2）事业单位□ （3）企业单位□ （4）务农□

（5）自由个体□ （6）社会团体□ （7）无业&其他□

9、您的平均年收入：

（1）3万及以下□ （2）4-6万□ （3）7-9万□ （4）10-12万□

（5）13万及以上

***问卷结束，感谢您的支持！***
